# Editorial: Editorial for mechanical and structural phenotypes of cells and extracellular matrices govern cell adhesion and migration

**DOI:** 10.3389/fcell.2023.1256311

**Published:** 2023-07-28

**Authors:** Claudia Tanja Mierke

**Affiliations:** Faculty of Physics and Earth Science, Peter Debye Institute of Soft Matter Physics, Biological Physics Division, Leipzig University, Leipzig, Germany

**Keywords:** mechanical characteristics, zyxin, PINCH-1, actin, random walk, metastasis, extracellular matrix (ECM), stiffness

The Research Topic on the mechanical and structural features of cells and their ECM surroundings is timely, as many articles have shown a bidirectional interplay between cells and their matrix environment (Findley et al., 1976; Mierke, 2014; Ruprecht et al., 2015; Mierke, 2020; Mierke, 2021). Recently, the mechanical characteristics of cells and their environment have been revealed to impact cell adhesion, migration and invasion (Almeida et al., 2005; McKenzie et al., 2018; Mierke, 2019; Espina et al., 2022; Takemoto et al., 2023). The Research Topic encompasses 27 articles, whereby the composition is very special, as there are a majority of 23 original research articles that cover physical, cell biological, mechanobiological and medical (oncological) fields, two reviews, one mini-review and one hypothesis and theory article. The Research Topic of articles covers various fields, such as the impact of cell mechanical characteristics on cell function (Brookes et al.; Bull et al.; Yao and Li), the effect of the environmental mechanical cues on cellular function (Chen and Qin; Pérez et al.; Yip et al.; Cai et al.; Chakraborty et al.; DeCastro et al.; Friedland et al.; Molter et al.; Pajic-Lijakovic et al.; Qin et al.; Ruiz-Franco and Van Der Gucht; Schaeffer et al.; Jetta et al.), the effect of mechanical cues on angiogenesis (Zapp et al.; Flournoy et al.), the transition of cells on their locomotion (Moldenhawer et al.; Nagle et al.), the impact of myofibroblast transdifferentiation of keratocytes on cell migration and sensitivity toward mesoscale curvatures (Van Der Putten et al.) and molecular contributions on (trans)migration and invasion (Niu et al.; Abdul Khaliq et al.; An; Baltes et al.; Cowan et al.; Mierke et al.).

Similar to cancer cells, stem cells, such as mouse embryonic stem cells (ESCs), can vary the expression of pluripotency genes together with biomechanical characteristics (Brookes et al.). Numerous mechanisms regulating cell shape and mechanical properties in somatic cells are known, but these mechanisms have not yet been explored in pluripotent cells. The authors did not observe any apparent correlation of REX1-GFP expression with cell spreading morphology as governed through circularity, Feret ratio, phase contrast brightness, or cell spreading area in their original article. The outcome was independent of whether the data were evaluated based on the individual parameters or using a combined metric obtained through principal component analysis from the four individual parameters. In addition, cell volume has not been seen to correlate with Rex1-GFP expression. A subpopulation of cells that can be easily dislodged by gentle shaking, can exhibit higher overall expression of Nanog and lower expression of LmnA, and indicates that enhanced expression of pluripotency genes may correspond to lower adhesion to the substrate, was not encountered. A relationship between cell stiffness and Rex1-GFP reporter expression has been detected using atomic force microscopy (AFM) and quantitative fluorescence imaging. Cells with high Rex1-GFP content uniformly display fairly low stiffness, whereas cells with low Rex1-GFP content exhibit a tendency toward higher stiffness scores. Finally, there is a certain interplay between pluripotency gene expression and biomechanical features. However, there also appears to be a strong role for other interactions between the cell culture regime and cellular biomechanical properties that sustain pluripotency regardless of the central transcriptional network.

In their original article, Bull and coworkers investigated whether actin dynamics can act as a multiscale integrator of cellular orientation supports during cell migration (Bull et al.). They explored how cells can set priorities among the cues. In specific, they investigated the integration of external cues in cellular cytoskeletal activity by observing the reaction of wavelike actin polymerization dynamics, which is the driver of cell motility, to electric field and nanotopography combinations in neutrophil cells. The electric fields present a general orientation and correspond closely to the *in vivo* situation at the wound locations. The nanotopographies resemble the dimensions of collagen fibers and serve as a local esotactic landmark. They found that cells favor guidance cues, with electric fields dominating long-term motility by emanating a unidirectional distortion of the sites where actin waves originate. This distortion rivals the wave guidance caused by the bidirectional nanotopographies. The conclusion of the authors was that actin wave properties provide a potent organizing principle for gaining an insight into cell behavior in intricate microenvironments.

While there is ample of knowledge on actin-driven cell migration and how forces can contribute to cell motility, Yao and Li reported in their original article an effective generation and transmission of forces in the course of mammalian cell movement (Yao and Li). Because cells *in vivo* are subjected to challenging physical environments with multiple barriers, gaining knowledge of the forces generated by cells will shed light on how cells cope in demanding circumstances. In this work, the authors utilized theoretical models to address actin- and water-driven cell migration and the impact of cell shapes on the generation of force. In specific, a multimodular mathematical approach has been employed to help in quantifying the effective force generated in actin- and water-driven cell migration. The findings reveal that the effective force produced by actin-driven cell migration is linear proportional to the amount of actin polymerization and the strength of FAs. The energy originates from actin polymerization counteracting the pressure of the actin network. The effective force produced from water-driven cell migration is related to the solvent active flow rate and the coefficient of external hydraulic drag. The energy inputs originate from active pumping of the solute counter to its concentration gradient. Their theoretical model predicts that the spatial distribution of the actin scaffold is mechanosensitive and the presence of globular actin is instrumental in imposing a biphasic cell speed in the strength of the focal adhesion (FA). Cell speed and effective force production also rely on cell shape induced by the intracellular actin flow field. Finally, model prediction gains knowledge on force production in biological and mechanobiological processes.

In their original article, Pérez and colleagues described an outside-in switch in integrin signaling elicited in reactive astrocytes in response to chemical and mechanical stimuli (Pérez et al.). Astrocyte reactivity is related to an impaired ability to heal the brain after injury when chemical or physical modifications occur in the damaged region. Tissue damage caused elevation of integrins and liberation of pro-inflammatory and extracellular matrix (ECM) proteins, which finally fosters the generation of a stiffer matrix environment. Thereby, the function of integrins is crucial, as they manifest a reactive phenotype within astrocytes, and the authors have been seen that α_V_β_3_ integrin couples to the Thy-1 (CD90) neuronal glycoprotein, which leads to elevated contractility and migration of astrocytes. In an alternative, α_V_β_3_ integrin perceives mechanical forces produced by the enhanced stiffness of the ECM. To date, the relationship between the α_V_β_3_ integrin mechanoreceptor reaction in astrocytes and the shifts in their reactive phenotype is ambiguous. To investigate the responsiveness to a combination of chemical and mechanical stress, astrocytes underwent excitation with Thy-1 protein A-coated magnetic beads and were subjected to a magnetic field to apply a mechanical stress. They examined the consequences of such stimulation on cell adhesion and contraction by analyzing the traction forces and their implications for cell morphology and integrin expression on the surface. Mechanical loading hastened the astrocyte reaction to the activation of integrin receptors through Thy-1, leading to cell adhesion and contraction. Astrocyte contraction subsequently exerted traction forces on the ECM that produced increased cell contractility at a faster pace and higher traction forces compared with Thy-1 on its own. Consequently, cell-external chemical and mechanical cues govern the responsiveness of astrocytes through initiating integrin upregulation, ligation, and behavioral signaling cues that increase cell contraction (mechanotransduction through an outside-in switch). These alterations in turn generate cell-intrinsic signals via actin-myosin engagement that elevate traction forces imposed onto the surrounding ECM (inside-out), which causes elevated ECM deformation and subsequently generation of astrogliosis. This work unveils the α_V_β_3_-integrin mechanoreceptor as a new target to modulate the deleterious impact of reactive astrocytes in neuronal healing. Altogether, the findings of the present work corroborate the idea of cellular tensegrity advocated by Ingber (Ingber, 2003a; Ingber, 2003b), where cells not merely perceive the mechanical cues of their microenvironment, but also produce mechanical inputs that are transferred to the ECM and can reshape the environment of the cells. Finally, a more rigid matrix encourages astrocyte reactivity and reduces the probability that neurons will recover from injury.

Yip and coworkers presented in their original research article how zyxin contributes to mechanosensation of stiffness and mechanotaxis behavior (Yip et al.). FA structures allow cells to perceive the stiffness of their ECM and convey these cues to the cell interior, causing restructuring of the actin cytoskeleton, maturation of FAs, and cell locomotion. Cells are recognized to move to areas of higher substrate stiffness, a phenomenon referred to as durotaxis. However, the fundamental molecular mechanism of durotaxis and the manner in which various proteins in the FA are involved are still elusive. Zyxin is a constituent of FA that is considered a link between the actin cytoskeleton and FA. They showed that knocking down zyxin compromised the capacity of NIH3T3 fibroblasts to perceive and react to alterations in the ECM, in regard to their FA sizes, the amount of cell tension, and the organization of F-actin. The rate of cell migration of fibroblasts with zyxin knockdown has also been unrelated to the accompanying underlying substrate stiffness, in opposite to wildtype fibroblasts, which migrated quickest at an average substrate stiffness of 14 kPa. Wildtype fibroblasts displayed durotaxic behavior on polyacrylamide gels with substrate stiffness gradients toward regions of growing substrate stiffness, whereas zyxin knockdown fibroblasts did not show durotaxis. The authors therefore concluded that zyxin is an essential protein necessary for stiffness and durotaxis recognition through regulation of FA sizes, cell traction stress, and architecture of F-actin.

DeCastro and colleagues explored in their original research article (DeCastro et al.) the individual organotrophic phenotype of brain- and bone-seeking MDA-MB-231 human triple negative breast cancer cells (TNBCs). Breast tumor cells that seek out brain have been shown to exhibit a perturbed proteomic profile that manifests in alterations of cell signaling pathways, cell cycle, metabolism, and restructuring of the ECM. Considering the unique microenvironmental features of brain and bone tissues, the authors hypothesized that TNBC cells (MDA-MB-231) targeting brain (MDA-BR) or bone (MDA-BO) tissues may display modified morphological or migratory phenotypes that deviate from that of parental TNBC cells (MDA-P), according to the biochemical or mechanical contexts of the microenvironment. The authors quantified cellular features including cell shape morphology, migration on 2D substrates, and stiffness in reaction to signals that to some extent imitate their ultimate metastatic cavity. Thereby, they detected that MDA-BO cells exhibit a specific protrusive morphology that is absent in MDA-P or MDA-BR. In addition, the authors reported that MDA-BO cells migrate stronger when cultured on collagen I, which is plentiful in bone tissue, compared with fibronectin, which is plentiful in brain tissue. Finally, MDA-BO cells exhibited larger FAs at higher densities in comparison to the other two cell subtypes. These findings will be used to establish a quantitative profile of mechanobiological phenotypes in TNBC that will be used in the future to help forecast the propensity for metastasis to organ-specific locations in a routine medical setting.

In their timely mini-review, Pajic-Lijakovic et al. presented the role of matrix viscoelasticity on collective cell movement on collagen I scaffolds (Pajic-Lijakovic et al.). Collective migration is a hallmark of many biological physiological processes, such as development, tissue homeostasis, and disease, such as cancer metastasis. As epithelial carcinomas invade, clusters of cells traverse the ambient stroma, which consists primarily of collagen I fiber meshworks. There is mounting scientific evidence that the rheological and topological characteristics of collagen networks can influence cell fate and the dynamics of cell migration. Cells apply mechanical forces to their medium as they migrate, leading to active reconstruction of ECM webs, which relies not solely on the forces generated, but also on the molecular pathways that dictate the rheology of the web. One facet of the rheology of collagen reticulations that arises as a critical determinant of cell performance is the viscoelasticity of the reticulation. The viscoelastic response of collagen I networks encountered in collective cell migration is governed by multiscale molecular processes that operate on multiple time scales. Cellular mechanical forces are adequate to effect structural alterations in collagen reticulations and can result in rheological alterations such as stiffening and residual stress accumulation, which are a function of the size and nature of the load and the rate of load variation. In collagen meshes, these impacts are governed principally through modifications in filament mobility occurring within the mesh. Dynamic rearrangement of ECM scaffolds can trigger local alterations in both network organization and mechanics, which in turn can affect the dynamics of cell migration and intercellular rearrangement. The structural alterations of collagen networks that occur in reaction to mechanical forces, and the importance of the role of collagen rheology and topology in governing cell behavior, are well established. In this mini-review, the authors examined the cause-and-effect correlation between the viscoelasticity of collagen networks and cell relocations at various spatial and temporal scales. Concentrating on structural alterations of collagen I networks encountered throughout collective cell migration, they focus on major rheological parameters and, in specific, on the contribution of viscoelasticity to local matrix stiffening observed in cell movement, which can induce alterations in cellular dynamics. Besides cell-based modifications in collagen networks, the rheological features of collagen structures can also affect cell performance, especially throughout single-cell and collective migration. Experimental alteration of network rheology presents new avenues to examine the interaction of cell-matrix relationships.

In their original research article, Cai and co-authors examined the compressive stress that promotes adhesion-dependent unjamming transfers occurring within the migration of breast cancer cells, such as 4T1 cells and MCF10A control cells (Cai et al.). The event of cellular unjamming refers to the collective fluidization of cell movement and has been implicated in multiple biological events, among them development, healing of wounds, and tumor growth. In cancer growth, the unregulated proliferation of cancer cells in a restricted area creates a mechanical compressive stress. Since various cellular and molecular mechanisms may be active simultaneously, though, the contribution of compressive stress in the decongestion of junctions encountered in cancer growth is still obscure. Therefore, the authors investigated what mechanism prevails in a tight, mechanically stressed monolayer and simulated it by employing a self-propelled Voronoi (SPV) model. They found that long-term mechanical pressure induces cell stalling in benign epithelial cells and promotes cancer cell migration at transitions that correspond to cell form, which led them to investigate the part played by cell-cell adhesion and substrate traction in unjamming transitions. They revealed that cadherin-based cell-cell adhesion governs the various cellular reactions to pressure stress and is an essential factor for unjamming in stressed monolayers. Compressive stress alone cannot trigger epithelial-mesenchymal transition in unjammed cells. In addition, traction microscopy shows mitigation of tensile stress in compressed cells inside the monolayer regardless of cell type and motility. While traction within the monolayer declines with compression pressure, cancer cells at the anterior edge of the cell layer show persistent traction in compression. Finally, strengthened intercellular adhesion and attenuation of traction forces within the bulk cell sheet under compression lead to fluidization of the cell layer and may impact collective cell motion in tumor development and breast cancer progression.

Motor axons in the chicken embryo that trace soft trails through evolving somite segments are examined in their original research article by Schaeffer and coworkers. During the development of the peripheral nervous system, motor axons successively sprout from the neural tube in a segmented manner to assure the functional integration of motor origins between the encircling cartilage and bone of the evolving vertebrae. This segmented sprouting is governed by the intrinsic characteristics of each segment bordering the neural tube (somite), in specific through chemical repulsive cues that are expressed in the posterior moiety. Nevertheless, knockout models for such repellent signals still show initial segmentation of sprouting motor axons, indicating the existence of complementary, unknown regulatory mechanisms for the segmentation of axon growth. Because neuronal growth is governed not solely through chemical cues but also through mechanical cues, the authors have analyzed the mechanical milieu of the outgrowing motor axons. Using AFM measurements on somite streaks of chicken embryos, in each segment a stiffness gradient prior to the growth of motor axons has been found. Axon growth has been confined to the anterior, softer tissue, which exhibited lower cell body density compared with the repulsive, stiffer posterior parts at subsequent developmental stages. In addition to well-known tissue stiffness regulating axon growth during development, the authors concluded that their findings suggest that motor axons also react to periodic stiffness gradients due to the intrinsic mechanical characteristics of somites.

Force transmission within disordered fiber networks has been studied in a research article by Ruiz-Franco and van der Gucht, who used a modeling approach (Ruiz-Franco and van der Gucht). In living tissues, residing cells exert forces toward their ambient environment to induce remodeling of ECM fibers and to transduce mechanical cues toward nearby cells. Therefore, they employed a reductionist model to examine how these forces, which act regionally through cell contraction, spread through the fibrous reticulum inside the ECM. As this network is highly heterogenous, continuum theories cannot be applied. They determined the properties of the ECM by dissecting how the propagation of forces is affected by the connectedness of the reticulation and the bending rigidity of the fibers. In highly interconnected fiber systems, stresses are distributed isotropically around the cell across a range that initially rises with growing contraction of the cell and then goes into a state of saturation at a characteristic distance. With lower degrees of connectivity, conversely, the stress pattern is highly asymmetric and typified by strings of forces that can propagate stresses over extremely long paths. Finally, the authors expected that their study of force transmission in fibrous networks can provide a new pathway for future investigations of how the mechanical loop feedback between the cell and the ECM is linked to the nanoscale cellular milieu.

How prostate cancer cells with raising metastatic potential exert different contractile forces, cell stiffness, and motility depending on the stiffness of the microenvironment has been examined by Molter and coworkers in their original research article (Molter et al.). In the course of malignant progression of cancer, such as metastasis, cancer cells face the challenge of moving through a crowded multicellular surrounding. At the same time, cancer cells seem to alter their biophysical characteristics. Cell softening and elevated contractility are seemingly omnipresent markers of metastatic advancement that may ease metastasis. Both cell stiffness and contractility are impaired through their surroundings. Stiffer matrices that closely mimic the tumor microenvironment tend to increase the contractility of metastatic cells, which fosters more contractile tumorigenic phenotypes. Prostate cancer (PCa), nevertheless, seems to deviate from these general cancer biophysical trends, as aggressive metastatic PCa cells seem stiffer instead of softer in comparison to their low-metastatic PCa pendants. While it has been published that metastatic PCa cells are more contractile compared to healthy cells, it has not been clear so far as to how cell contractility alters with advancing metastatic potency of PCa. Biophysical alterations of PCa cells with increasing metastatic potential, such as a panel of progressively increasing metastatic potential cell lines (22RV1, LNCaP, DU145, and PC3) that depend on the stiffness of the microenvironment have been examined by the authors. Therefore, the contractilities of the cell lines have been determined using traction force microscopy (TFM), cortical stiffness using optical magnetic twisting cytometry (OMTC) and cell motility employing time-lapse microscopy. They revealed that PCa contractility, cell stiffness, and migration do not universally correlate with metastatic capacity. Instead, PCa cells with different metastasis potency display unique biophysical reactions that are influenced variably through substrate stiffness. Notwithstanding this biophysical multiplicity, this work infers that the mechanistic microsite is a pivotal determinant of the biophysical reaction of PCa with different metastatic potency. The mechanistic focus and methodology of the investigation are somewhat singular and complement traditional biochemical and genetic approaches that are commonly used to gain an insight into this disease.

Friedland and colleagues investigated in their original research article how ECM-based shear stress triggers extrusion of apoptotic cells during early mammary gland evolution (Friedland et al.). Epithelial cells of human mammary glands are subjected to different mechanical ECM stresses that govern tissue formation and homeostasis. The mechanical adaptation of mammary gland tissue to shear stress transmitted by the ECM has been insufficiently explored because of the absence of robust experimental approaches. Thus, the authors constructed a magnetic shear strain device that allowed them to dissect, for the first time, the instantaneous shear strain reaction of human mammary gland cells. MCF10A-derived mammary acini with basement membranes of specified maturation condition and basoapical polarization have been utilized to mimic mammary gland morphogenesis *in vitro*. With novel biophysical testing instrument, living spheroids incorporated in an ultra-soft matrix of below 60 Pa have been subjected to cyclic shear stress with defined amplitudes (≤15%, 0.2 Hz) for multiple hours. They found that mammary spheroids exhibited higher shear strength, which enhanced with basement membrane maturation and basoapical polarization. Interestingly, underdeveloped spheroids tended to extrude apoptotic cells from the spheroid body, triggered by cyclic stresses. In juxtaposition, mature spheroids have been impervious to this mechanoreaction which is an indication of modified mechanosensing or mechanotransduction mechanisms occurring during the morphogenesis of mammary tissues. Subsequently, a multifaceted instrument for investigating the reaction of 3D cell culture models to cyclic shear stress has been presented. It can in theory be utilized for all types of cell clusters, including those that are grown exclusively in ultra-soft hydrogels. It appears that this type of approach holds promise for providing new insights into dynamic, shear-induced mechanobiological control cycles between cells and their ambient ECM.

In another original research article Qin and co-workers explored how the tumor suppressor DAPK1 facilitates adhesion formation on rigid matrices, while causing anoikis on soft matrices (Qin et al.). Cancer cells usually grow on soft surfaces because the mechanosensitivity of the ECM is disturbed. When mechanosensitivity is reinstated, cancer cells undergo apoptosis (anoikis) as most normal cells undergo on soft surfaces. The connection between mechanosensation and activation of anoikis, nevertheless, has not been clarified. The authors demonstrated that death-associated protein kinase 1 (DAPK1), a tumor suppressor enabling cell death, is directly coupled to anoikis activation via stiffness sensing. They established that cells compromised in their stiffness sensitivity due to obstruction of DAPK1 activity are converted for growth on soft matrices. In addition, DAPK1 facilitates the catalysis of matrix adhesion establishment and is involved in adhesion to stiff surfaces. This pathway entails DAPK1 phosphorylation of tropomyosin1.1 and the talin1 head domain, as well as Src-mediated tyrosine phosphorylation of DAPK1. On soft surfaces, DAPK1 quickly detangles from adhesion complexes and initiates apoptosis catalyzed through PTPN12 activity and the talin1 head. Consequently, DAPK1 is critical for adhesion formation on rigid surfaces and for anoikis activation on soft surfaces by its attachment to modules that perceive stiffness.

Jetta and colleagues examined in their original research article that epithelial cells, such as Madin-Darby Canine Kidney (MDCK) cells, perceive local stiffness through Piezo1-driven cytoskeletal restructuring (Jetta et al.). The stiffness of the local substrate is an important mechanical factor in tissue organization throughout its development and rearrangement. It is well established that adherent cells exploit transmembrane proteins, such as integrins, at FAs to transduce mechanical signals of the ECM into intracellular biological events. The authors showed that epithelial cells react to substrate stiffening mainly through rearrangement of the actin cytoskeleton, which entails activation of mechanosensitive Piezo1 channels. Cells in which Piezo1 has been knocked down abolished actin stress fibers that built up on stiff substrates, whereas the effects on cell morphology and spreading area remained minimal. Attenuation of Piezo1 channels with GsMTx4 also substantially diminished the stiffness-induced rearrangement of F-actin, which suggests that cation current conveyed through Piezo1 is involved. Piezo1 channel activation by a specific agonist, such as Yoda1, thickened F-actin fibers and expanded FAs on stiffer substrates, while nascent FA generation, which eases spreading on soft substrates, remained undisturbed. These results show that Piezo1 acts as a force sensor that connects to the actin cytoskeleton to discriminate the rigidity of the substrate and function to aid in adaptive restructuring of the epithelium.

In their original research article Chen and Qin developed a coarse-grained random walk model to explore the dynamics within a cell (Chen and Qin). Cell migration across the ECM is pivotal for multiple physiological processes including tissue development, immunological reactions, and cancer metastasis. Traditional models including persistent random walk (PRW) and Lévy walk account for the migratory dynamics of only certain cell types in a homogeneous ambient environment. More recently, it has been revealed that intracellular actin flux can ensure universal coupling between cell migration velocity and persistence for a multitude of cell types moving in *in vitro* assays and within living tissues (Maiuri et al., 2015; Mierke, 2019; Thüroff et al., 2019; Wortel et al., 2021). The implications of the speed-persistence relationship on macroscopic cell migration dynamics and patterning in intricate environments, nonetheless, are widely obscure. In their model the cell is treated as a simplified dimensionless particle that migrates through homogenous and porous matrices with correlated velocity and endurance (persistence). Thus, the authors designed a Monte Carlo random walk simulation to investigate the locomotion, search capability, and search effectiveness of a cell locomoting in both homogeneous and porous surroundings. The motility, such as MSDs and distributions, the search capability, such as the number of objectives detected in a specific time period, and the search efficiency, such as the number of objectives detected per unit distance, are computed and benchmarked against the four motion tactics, such as the Lévy walk, persistent random walk (PRW), random walk with linearly correlated speed and persistence (linear RWSP) and random walk with nonlinearly correlated speed and persistence (nonlinear RWSP). Coarse-grained analysis revealed that the nonlinear RWSP attained the highest motility in both homogeneous and porous settings. When a particle is hunting for targets, the nonlinear coupling of velocity and persistence enhances search efficiency, which means that more targets are encountered in a certain period of time, while sacrificing search efficiency, which means that fewer targets are encountered per unit distance. In addition, both convex and concave pores constrain particle movement, notably in the nonlinear RWSP and Lévy walk. In summary, the findings show that the nonlinear correlation of velocity and persistence has the power to enhance motion and search characteristics in complex environments and could be used as a starting point for more in-depth investigations of active particles in biological, engineering, and social science disciplines.

In their original research article, Chakraborty and coworkers examined the effects of early tension adjustment in conjunction with the surface myomerger required for primary fusion of C2C12 myoblasts (Chakraborty et al.). The authors investigated the time-dependent control of fluctuating tension during myogenesis and the role of the fusogen myomerger. They determined nanometric height fluctuations of the basement membrane of C2C12 cells following initiation of differentiation. Fusion of cells raises the fluctuating tension but preferentially causes a transient drop in tension at a few or several hours. Cells fail to merge when the early tension is steadily enhanced through methyl-β-cyclodextrin (MβCD). Disruption of tension regulation decreases levels of fusion as well. Throughout this pre-fusion phase, cells that ultimately differentiate generally exhibit reduced tension compared to other non-fusing cells, confirming that early tension states are associated with the fate choice. Early tension decrease is paralleled by a low, but progressively rising, surface myomerger concentration level. Regionally, areas of higher myomerger content also exhibit lower tension. This negative correlation, nevertheless, is completely dissipated in the early phase by MβCD-derived cholesterol depletion or later with progressive differentiation. The authors revealed that as tension and surface myomerger accumulation increased at these settings, the myomerger clusters diffused substantially. They concluded that low tension involving early-phase surface myomerger clusters is vital for fusion and can be perturbed with cholesterol-lowering molecules, which has the inherent potency to impact muscle health.

In the following two manuscripts present the effect of mechanical signals on angiogenesis. In 3D angiogenesis models, Zapp and coworkers presented in their original research article that the natural presentation of glycosaminoglycans (GAGs) in synthetic matrices is crucial (Zapp et al.). GAGs comprise long, linear polysaccharides that are present in the ECM of higher organisms and are bound either covalently to protein kernels, as proteoglycans, or in free state. According to their chemical makeup and structure, GAGs control a broad spectrum of essential roles in tissue homeostasis. In line with this, GAG-based biomaterials have an important part to fill in tissue engineering. Contemporary biomaterials utilize cross-links between chemically modified GAG chains. Due to alterations along GAG chains, GAG-protein interactions and accessibility are restricted to unravel the biochemical and biophysical features that regulate GAG functions. In this case, a natural appearance of GAGs is obtained through terminal immobilization of GAGs on a polyethylene glycol (PEG) hydrogel. Physicochemical typing revealed that various end-thiolated GAGs can be integrated within physiological levels of concentration, while the mechanical characteristics of the hydrogel can be adjusted solely through PEG polymer concentration. The functional benefit of this assay has been exemplified in a 3D cell culture setting. Immobilization of end-thiolated hyaluronan promoted the generation of capillary-like sprouts emanating from embedded endothelial cell spheroids. In summary, the presented PEG/GAG hydrogels establish an indigenous microenvironment with precisely adjustable mechanobiochemical characteristics and are an efficacious instrument to investigate and harness the bioactivity of GAGs.

Flournoy and colleagues explored the mechanical control of signal transduction in the process of angiogenesis in their review (Flournoy et al.). Biophysical and biochemical cues cooperate to regulate angiogenesis. These cues govern angiogenesis in the course of developmental processes and wound healing circumstances. Abnormal signaling contributes to pathological angiogenesis occurring in cancer advancement. In their review, the authors summarized the known signaling pathways involved in mechanotransduction, which is critical for angiogenesis. They discussed how mechanical microenvironment variances in stiffness, ligand accessibility, and topography can modify the angiogenesis process. In addition, the authors presented an integrated perspective on how mechanical disturbances like stretch and fluid shear cause the angiogenesis-related signal transduction to be acutely modified, which results subsequently in downstream gene expression. Tissue engineering methods to examine angiogenesis are also included for discussion. Future directions are suggested to support efforts to unmask a global portrait of angiogenesis.

The following two original research articles address the transition of cells on their movement. Moldenhawer and coworkers describe the spontaneous transitions between amoeboid and keratocyte-like cellular migration modes (Moldenhawer et al.). It is well established that the motility of adherent eukaryotic cells is fueled through the dynamical remodeling of the actin cytoskeleton. Although there exists a general force-generating actin mechanism, various cell types frequently exhibit distinct modes of movement that are divergent in shape dynamics, velocity, and persistence of migration. Recently, it has been revealed that several modes of movement can be initiated in the model organism *Dictyostelium discoideum*, as a function of genetic alterations, developmental constraints, and synthetic alterations in intracellular signaling. The authors found that in a mutant *Dictyostelium discoideum* cell line with enhanced Ras activity, spontaneous switching between two distinct modes of migration, the amoeboid and fan-shaped locomotion types, took place even within the same cell. The authors detected and characterized that there are repeated and reversible shifts between the two modes of locomotion, indicating that different behaviors are present in the selfsame cell. Consequently, they modified a traditional phenomenological motility model by coupling a reaction-diffusion system for intracellular dynamics with a dynamic phase field to account for their experimental findings.

Nagle and colleagues examined the surface tension of model tissues in the course of transformation of malignancy and epithelial-mesenchymal transformation (Nagle et al.). The well-established epithelial-mesenchymal transition and its hybrid-states are implicated in migration, invasion, and metastasis. The transfer of these alterations to the tissue scale has not yet been addressed, despite being crucial for the comprehension of cancer propagation. Therefore, biophysical instruments for measurements in model tumor systems are required to unravel the impact of epithelial-mesenchymal transition at the collective cellular level. Employing an established biophysical approach relying on the incorporation of magnetic nanoparticles into cells, the authors have formed and planarized multicellular assemblies to examine the implications of the depletion of the metastasis suppressor NME1 on the mechanical characteristics at the tissue level. Multicellular spheroids act as viscoelastic fluids, and their equilibrium form is governed through surface tension, which is determined through their deformation when a magnetic field is exerted. In a model of breast cancer cells genetically edited for NME1, the authors correlated tumor invasion, migration, and adhesion alterations with shape-retaining characteristics by gauging surface tension and investigating both invasion and migration potency, as well as adhesion characteristics.

Van der Putten and coworkers explored the myofibroblast transdifferentiation of human corneal keratocytes that impact migration and perception of mesoscale curvatures in their original research article (Van Der Putten et al.). Functional tissue repair after damage or disease is governed through the regenerative or fibrotic reaction of the cells in the tissue. In the setting of corneal impairment, keratocytes are an important cell type that dictates the result of the remodeling reaction by adjusting to either a fibroblast or myofibroblast phenotype. Even though a burgeoning amount of literature indicates that geometric characteristics in the environment can affect the myo(fibroblast) phenotype, there is a shortage of information on whether and how the differentiated keratocyte phenotype is impacted through curved tissue geometry in the cornea. To fill this gap, the authors characterized the phenotype of fibroblastic and transforming growth factor β (TGFβ)-induced myofibroblastic keratocytes and examined their migratory characteristics on curved culture substrates with varying levels of curvatures. Immunofluorescence imaging and quantification of cell morphologic characteristics revealed that fibroblastic keratocytes seemed to be commonly more elongated, while myofibroblastic keratocytes possessed more prominent α-smooth muscle actin (α-SMA), actin stress fibers, and more mature focal adhesions. Intriguingly, keratocyte adhesion remained weak and non-stable on convex structures, whereas they adhered normally on flat and concave surfaces. On concave cylinders, fibroblastic keratocytes moved longitudinally more rapidly and with higher persistence as compared to myofibroblastic keratocytes. In addition, this response appeared more prominent in smaller cylinders (i.e., larger curvatures). In summary, both keratocyte phenotypes can perceive and react to the sign and magnitude of substrate curvature. Nevertheless, myofibroblastic keratocytes display poorer curvature perception and more gradual migration on curved substrates compared with fibroblastic keratocytes. These results provide essential glimpses into the phenotype of keratocytes after wounding, but also highlight the power of attuning the physical cellular surroundings in tissue-engineering approaches to guide a beneficial regenerative outcome.

The following six articles (four original research articles, one hypothesis and theory article and one review) deal with the molecular contributions on (trans)migration and invasion. In their original article, Niu and colleagues examined HIV Tat-driven induction of monocyte transmigration at the blood–brain barrier (BBB), whereby the function of the chemokine receptor CXCR3 is in the focus (Niu et al.). HIV transactivator of transcription (Tat), one of the cytotoxic proteins secreted by HIV-infected cells, is also recognized to enhance chemokine-mediated migration of monocytes toward the brain, which in turn causes neuroinflammation, thereby participating in the generation of HIV-associated neurocognitive disorders (HAND). However, the mechanisms underpinning HIV Tat-driven amplification of monocyte migration were mostly unidentified. It is well-established that CXC chemokine receptor 3 (CXCR3), which is expressed by peripheral monocytes, is involved in the influx and accumulation of monocytes. The authors demonstrated that exposure of human monocytes to HIV Tat protein results in enhanced expression of CXCR3, which in turn causes enhanced transmigration of monocytes through the BBB in both *in vitro* and *in vivo* model environments. This process entailed activation of Toll-like receptor 4 (TLR4) with subsequent phosphorylation and activation of TANK-binding kinase 1 (TBK1) and followed by phosphorylation and nuclear translocation of interferon-regulatory factor 3 (IRF3), which finally caused enhanced expression of CXCR3 in human monocytes. These findings point to a new type of molecular mechanism underpinning the HIV Tat-based enhancement of monocyte migration by the BBB while proposing a novel role for CXCR3-dependent monocyte movement in HIV Tat-based neuroinflammation.

Khaliq and coworkers revealed in their original research article that the C-peptide facilitates cell migration through regulation of matrix metallopeptidase-9 (MMP-9) activity by direct modulation of β-catenin in human endometrial stromal cells (ESCs) (Abdul Khaliq et al.). The ESC motility participates in the recovery of the functional layer of the endometrium and consequently assists trophoblast invasion throughout early pregnancy. Following differentiation of ESCs through decidualization in reaction to progesterone occurring in the menstrual cycle and embryo implantation, decidualized ESCs (D-ESCs) exhibit increased motility and greater invasive activity. Human proinsulin-linking peptide (C-peptide) is generated in equimolar quantities in the proteolysis of insulin within pancreatic β-cells. Nevertheless, the role of C-peptide in human endometrial cellular motility has not yet been addressed. Therefore, the authors pinpointed C-peptide as a detrimental factor in the migration of undecidualized human endometrial stromal cells (UnD-ESCs). C-peptide enhanced the migration and invasion of UnD-ESCs and trophoblast-derived Jeg3 cells and not ESCs after decidualization, which represents a functional and biochemical differentiation of UnD-ESCs. Akt and protein phosphatase 1 each control β-catenin phosphorylation in UnD-ESCs but not in D-ESCs, and consequently enhanced β-catenin nuclear translocation in C-peptide-treated UnD-ESCs. C-peptide has been found to enhance the activity of MMP9 through enhancing MMP9 expression and reducing the expression of metallopeptidase inhibitor 1 (TIMP1) and TIMP3. The expression of the three molecules can be controlled through direct binding of β-catenin in the regulatory region of their promoters. Attenuation of either β-catenin or MMP9 restrained C-peptide-increased migration in UnD-ESCs. In summary, these results imply that C-peptide content is pivotal in controlling the migration of UnD-ESCs, demonstrating the relationship between C-peptide concentration and the trophoblast invasion miscarriage rate through triggering abnormal migration in UnD-ESCs in hyperinsulinemia or polycystric ovary syndrome (PCOS) patients.

In their original research article, Mierke and colleagues examined the role of PINCH1 in the migration of fibroblasts in ECMs and how it impacts the cell mechano-phenotype (Mierke et al.). Cell migration plays a crucial role in multiple physiological processes, such as tissue homeostasis or healing of wounds after tissue injury, but also in pathological processes, which comprise malignant advancement of cancer. The efficiency of cell migration and invasion seems to be dependent on the cytoskeletal mechano-phenotype. The characteristics of the cytoskeleton rely on internal cytoskeletal and external environmental determinants. Connections between the cell and its local matrix microenvironment, established by cell-matrix adhesion receptors, are a contributing factor. When activated, FA proteins like PINCH1 are actively recruited to evolving FA sites. PINCH1 specifically interfaces with ILK, which couples to cell matrix receptors and the actomyosin cytoskeleton. Nevertheless, the function of PINCH1 in cellular mechanics governing cellular motility in 3D collagen matrices was uncertain. The authors hypothesized that PINCH1 drives 3D motility through governing cellular mechanical characteristics, such as stiffness. Therefore, PINCH1 wild-type and knock-out cells have been evaluated in for their capacity to migrate in dense extracellular 3D matrices. Actually, PINCH1 wild-type cells migrated more abundantly and more deeply in 3D matrices as compared to knock-out cells. In addition, cellular deformability has been assessed, such as the elastic modulus (stiffness). PINCH1 knock-out cells are more deformable (compliant) relative to PINCH1 wild-type cells. Migration of both PINCH1^−/−^ cells and PINCH1^fl/fl^ cells was diminished by the inhibition of actin polymerization with latrunculin A, suggesting that variations in the actin cytoskeleton are not accountable for their divergent invasiveness. Nevertheless, the mechanical phenotype of PINCH1^−/−^ cells might be reflected by latrunculin A treatment of PINCH1^fl/fl^ cells, as they display similar deformability to untreated PINCH1^−/−^ cells. Moreover, there exists a discrepancy between the long axis elongation and short axis contraction between PINCH1^fl/fl^ cells and PINCH1^−/−^ cells after latrunculin A treatment, which is mirrored by a change in the proxy values for their Poisson’s ratio. The authors concluded that this is likely due to changes in the architecture of the cytoskeleton. The nonmuscle myosin II inhibitor blebbistatin also decreased cell invasiveness in extracellular 3D matrices but stiffened cells instead. Finally, PINCH1 evidently is integral to the mechanical stiffness of cells due to the actin cytoskeleton, which governs 3D motility.

The stabilization of actin has been investigated by Baltes and coworkers in their original research article (Baltes et al.). The cytoskeletal filament actin is engaged in a wide range of biological tasks, such as shaping cells or producing and transmitting forces. Especially relevant for these functions is the capacity of actin filaments to elongate and shrink. To investigate the function of actin in living cells, the dynamical nature has to be specifically addressed. In the past, such modifications were achieved through destabilization of actin. In a completely different approach, the authors used the natural compound miuraenamide A in living retinal pigment epithelial cells (RPE-1) to stabilize actin filaments and demonstrated that it diminished actin filament dynamics and extended filament length. Miuraenamide A-treated cells enlarged their adhesion patch and displayed multiple FA sites. These changes lead to a reduced migration rate and a displacement of the nuclear location. The authors consider miuraenamide A to be a promising new compound for stabilizing actin polymerization that can be applied to examine cell behavior, like cell migration.

In a highly recognized hypothesis and theory article An delineated that MRTF could be the connecting piece in a multiscale mechanobiological approach to macrophage malfunction in space (An). Macrophages in space display compromised phagocytosis, adhesion, migration, and cytokine production, which hampers their capacity to trigger immune reactions. In view of the fact that the combined microgravity and radiation effects of spaceflight are multifaceted and multifactorial, it is to be anticipated that contradictory results are often encountered. Therefore, research findings on the macrophage responsiveness to spaceflight are revisited at various time scales, such as seconds to weeks, and spatial scales, such as at molecular, intracellular, extracellular to physiological tissue/organ level. Among the major results were the time-dependence of pro-inflammatory cytokine activation and integrin expression. The author presented the time-dependent intracellular locus of MRTF-A as a hypothetical perturbing factor of macrophage activation. She discussed the dependence of the mechanosensitive MRTF-A/SRF signaling on the actin cytoskeleton/nucleoskeleton, microtubules, cell surface mechanoreceptors, hypoxia, oxidative stress, and intracellular/extracellular crosstalk. By embracing a multiscale perspective, this work provides the first mechanistic answer to a three-decade-old question concerning compromised cytokine secretion in microgravity and reinforces the link among recent progress in mechanobiology, microgravity, and the immune response in spaceflight. Ultimately, she hypothesized MRTF participation and complications in the treatment of spaceflight-related cardiovascular, skeletal, and immune disorders.

In an intricate review article Cowan and colleagues discussed the role of non-muscle myosin II (NmII) and the plasticity of 3D cell migration (Cowan et al.). Constrained cells moving through 3D environments are also governed by the laws of physics, i.e., for every action there needs to be an like and contrary reaction for the cells to move. Cells can exploit multiple distinct molecular mechanisms for their locomotion, which is reflected in the multiple modes by which NMII can produce the mechanical forces necessary for 3D cell migration. The authors presented an overview that summarizes the unique 3D migration modes and revealed how NMII activity is coordinated and specialized in each of these distinct modes. In addition, the authors highlighted tropomyosins and septins as two protein families that are thought to provide more mystery about how NMII activity is controlled throughout 3D cell migration. Consequently, this information ultimately suggests that studying the mechanisms that govern NMII activity will be beneficial in gaining an improved insight into how a single cell alternates between various modes of 3D migration in reaction to the physical environment.

The unparalleled Research Topic of 27 articles illustrates the intricacy of the diverse types of methods and new branches of cell adhesion and migration research, such as the mechanobiological approaches. The biochemical and mechanical properties of cells, their matrix environments, cell assemblies, spheroids and tissues have been examined using a wide variety of methods. There is a clear movement from 2D models toward 3D molecules in the field of cellular motility. Universal underlying principles seem to be present in cell migration and adhesion that are discovered by intricate interdisciplinary approaches. Consequently, interdisciplinary research, such as biology, medicine, bioengineering, bioinformatics and physics is required for exploring the interplay between cells, assemblies of cells, tissues and their ambient environment. This also constantly requires the further development and new development of biophysical methods.
